# Classification of colorectal carcinoma subtypes based on ferroptosis-associated molecular markers

**DOI:** 10.1186/s12957-022-02575-5

**Published:** 2022-04-12

**Authors:** Qingfang Yue, Yuan Zhang, Fei Wang, Fei Cao, Xianglong Duan, Jun Bai

**Affiliations:** 1 Department of Medical Oncology, Shaanxi Provincial People’s Hospital, Affiliated Hospital of Northwestern Polytechnical University, Xi’an, Shaanxi People’s Republic of China; 2 Institute of Medical Research, Northwestern Polytechnic University, Xi’an, Shaanxi People’s Republic of China; 3 Department of Gynecology, Shaanxi Provincial People’s Hospital, Affiliated Hospital of Northwestern Polytechnical University, Xi’an, Shaanxi People’s Republic of China; 4 Second Department of General Surgery, Shaanxi Provincial People’s Hospital, Affiliated Hospital of Northwestern Polytechnical University, Xi’an, Shaanxi People’s Republic of China; 5 Medical college, Xizang Mingzu University, Xianyang, Shaanxi People’s Republic of China

**Keywords:** Colorectal carcinoma, Ferroptosis-related genes, Classification, Prognosis, Immune infiltration

## Abstract

**Background:**

Ferroptosis is associated with the development of many cancers; the molecular features of colorectal carcinoma (CRC) based on ferroptosis-related genes (FRGs) remain unknown. Herein, we aimed to identify ferroptosis-associated molecular subtypes of CRC based on the expression profiles of FRGs.

**Methods:**

To explore ferroptosis-associated subtypes of CRC, the gene expression data and clinical information of 682 patients were extracted from The Cancer Genome Atlas and Gene Expression Omnibus databases. We performed consensus clustering to identify robust clusters of patients. Then the distribution of the subtypes in terms of prognosis significance, transcriptome features, immune microenvironment, drug sensitivity, gene mutations, and copy number alternations (CNAs) were evaluated respectively. In addition, we analyzed the correlation of these ferroptosis-associated molecular subtypes with the distribution of conventional clinical indicators in CRC.

**Results:**

Four subtypes of CRC (C1, C2, C3, and C4) were identified in which the prognosis, immune cell infiltration, immune score, stromal score, and tumor purity were significantly different between the four subtypes. The C3 subtype had a higher infiltration of B cells, M2 macrophages, resting mast cells, monocytes, natural killer cells, plasma cells, and CD8 T cells. The C3 subtype had the highest immune and stromal scores and the lowest tumor purity. In contrast, the C4 subtype demonstrated the lowest immune and stromal scores and the highest tumor purity. Programmed cell death ligand 1 (PD-L1), an immune checkpoint protein, was differentially expressed in the four subtypes (*P* < 2e–16) and was significantly correlated with the expression of several FRGs in all subtypes. Significant differences in stem cell indices (*P* < 0.01) and drug sensitivity (*P* < 0.01) were observed in the four subtypes. Additionally, gene mutations analysis showed that FRGs such as *TP53* had a high frequency of mutation in the four subtypes (49%, 62%, 61%, and 71%, respectively), and the CNAs showed significant difference among all subtypes (*P* < 0.001).

**Conclusion:**

In summary, the ferroptosis-associated subtypes could serve as an independent biomarker for estimating oncological outcomes in patients with CRC. Our results demonstrated that the high level of heterogeneity in the expression of FRGs might be useful for the stratification of patients with CRC and the implementation of individualized therapeutic strategies.

**Supplementary Information:**

The online version contains supplementary material available at 10.1186/s12957-022-02575-5.

## Background

Colorectal carcinoma (CRC) is the third most commonly diagnosed cancer and is the second leading cause of cancer-related deaths globally [1]. Although current treatments such as targeted therapy, immunotherapy, and precision treatment have been applied for the treatment of CRC [2, 3], the clinical outcomes are unsatisfactory, and the prognosis of patients with CRC remains bleak. CRC is a heterogeneous disease [4], and the poor prognosis and relapse in patients with CRC are mainly attributed to its heterogeneity. The genetic and molecular characteristics of tumor cells determine their aggressiveness and susceptibility to treatment [5]. Therefore, it is crucial to integrate the study of molecular subtypes into the clinical management of CRC.

Recently, ferroptosis has been identified as a regulated form of cell death, which is characterized by the iron-dependent accumulation of lipid peroxides [6]. A plethora of studies have indicated that ferroptosis play an important role in carcinogenesis, and it is a promising target for the treatment of cancers, especially tumors that show resistance to conventional treatments [7, 8].

In recent years, the role of ferroptosis in CRC has attracted much attention. For example, Xie et al. demonstrated that TP53, a tumor suppressor, could inhibit the activity of dipeptidyl peptidase 4 in a transcription-independent manner, thus inhibiting erastin-induced ferroptosis in CRC [9]. Park et al. demonstrated that bromelain effectively induces higher levels of ferroptosis in KRAS-mutant cell lines than in wild-type KRAS cells by modulating ACSL4 levels [10]. Sui et al. revealed that GPX4 inhibition was a key determinant in RSL3-induced ferroptosis in CRC [11]. Xu et al. reported that targeting SLC7A11, a cystine/glutamate antiporter, is a potential strategy in the treatment of CRC; erastin inhibited SLC7A11 and selectively killed CRC stem cells by inducing ferroptosis and attenuating chemoresistance in CRC [12]. Although previous studies have identified several gene markers associated with ferroptosis in CRC, the relationship between ferroptosis-related genes (FRGs) and CRC treatment and prognosis is unknown.

In the present study, we first collected the mRNA expression profiles and corresponding clinical data of patients with CRC from public databases. Then, three cohorts were merged into a metadata set of 682 patients. We classified CRC into four distinct subtypes based on consensus clustering of FRG profiles. Furthermore, we evaluated the distribution of the four subtypes in terms of prognosis significance, transcriptome features, immune microenvironment, drug sensitivity, gene mutations, and copy number alternations (CNAs). Finally, we analyzed the correlation of these ferroptosis-associated molecular subtypes with the distribution of conventional clinical indicators in CRC. The identification of ferroptosis-related subtypes may provide important prognostic and therapeutic values in the diagnosis and treatment of patients with CRC.

## Materials and methods

### Data collection

RNA sequencing (RNA-seq) data and clinical information of patients with colon carcinoma (*n* = 461) and rectal carcinoma (*n* = 172) were downloaded from The Cancer Genome Atlas database (TCGA, http://cancergenome.nih.gov/). Additionally, gene expression data together with clinical information of 49 colon carcinoma samples in the GSE152430 dataset were downloaded from Gene Expression Omnibus (GEO, http://www.ncbi.nlm.nih.gov/). The clinical characteristics of patients obtained from the TCGA and GEO datasets are shown in supplementary Table S1. Raw counts of RNA-seq data were transformed into transcripts per kilobase million values for subsequent analysis. Next, three RNA-seq datasets were merged into a metadata set of 682 CRC samples, and the ComBat function in the “sva” package in R was applied to remove the batch effects [13]. Then, after batch effect correction, principal component analysis (PCA) was performed with the “princomp” function of the “stats” R package (Supplementary Fig. S1). The datasets from TCGA and GEO databases are both publicly available. The current study follows the TCGA and GEO data access policies and publication guidelines.

### Evaluation of FRG expression profiles in CRC

Expression profiles of 60 FRGs were obtained from previous studies [14–16] (Supplementary Table S2). Next, gene expression profiles of FRGs in CRC samples were extracted, and the candidate genes were used for clustering. The “RCircos” [17] package in R was used to characterize the distribution of FRGs in CRC samples.

#### Consensus cluster analysis to identify ferroptosis subtypes

Initially, consensus classification of CRC was performed in the metadata set using the “ConsensusClusterPlus” package in R, which offers stable quantitative and visual evidence for estimating the number of unsupervised clusters in a dataset [18]. In each algorithm, tumors were sampled 100 times, and the k-means clustering algorithm based on the Euclidean distance metric was performed. The clustering number was assessed according to the area under the cumulative distribution function (CDF) curve [19]. Next, the correlation of the subtypes with overall survival (OS) was analyzed by performing Cox regression analysis using the “survminer” package in R [20]. A heatmap was plotted according to the tumor/node/metastasis (TNM) stage of the samples.

In addition, the analysis of variance (ANOVA) test was used to screen the differentially expressed ferroptosis-related genes (DE-FRGs) among subtypes, and the P values were adjusted by Benjamini & Hochberg (BH) correction (*P* < 0.05). Then, Pearson’s correlation analysis was performed to analyze the correlation of ferroptosis-associated genes co-expression in CRC. Finally, PCA was performed to assess the subtypes based on the expression profiles of DE-FRGs using the “principal” function of the “psych” R package.

### Functional enrichment analysis

The “clusterProfiler” package in R was used to conduct Gene Ontology (GO) and Kyoto Encyclopedia of Genes and Genomes (KEGG) analyses based on the DE-FRGs in CRC [21]. Functional annotations were considered significantly enriched when the BH-adjusted P values were less than 0.05.

### Immune cell infiltration analysis

The “CIBERSORT” package in R was used to evaluate and compare the infiltration of 22 immune cell types among ferroptosis-associated molecular subtypes [22]. The immune score, matrix score, and tumor purity of the subtypes were calculated by the “ESTIMATE” package in R [23]. In recent years, cancer immunotherapy based on immune checkpoint inhibitors (ICIs) has achieved considerable success in the clinical setting. The expression profiles of programmed cell death ligand 1 (PD-L1), an immune checkpoint protein, and FRGs were extracted from the metadata set, and the differential expression of PD-L1 in CRC subtypes was determined. The “ggplot2” package in R was used to analyze the correlation between PD-L1 and DE-FRGs.

### Calculation of stemness index

The stemness index was calculated using an innovative one-class logistic regression (OCLR) machine learning algorithm, as previously described [24]. To construct the stemness index, data from stem cells and their differentiated ectodermal, mesodermal, and endodermal groups of cells were obtained from the Progenitor Cell Biology Consortium (https://www.synapse.org/pcbc) database as the initial dataset, followed by calculation of the stemness index using the one-class logistic regression (OCLR) algorithm. Then, the transcriptome expression profiles corresponding to the stemness index obtained from the OCLR-based calculation were applied to the metadata set to calculate the mRNA stemness index (mRNAsi) for each sample. By using a linear transformation, the mRNAsi was mapped to the range [0,1] by subtracting the minimum value and dividing it by the maximum value.

### Correlation analysis of drug sensitivity among subtypes

To identify potential compounds that might be effective against the CRC subtypes, the “pRRophetic” package in R was used to predict the drug sensitivity in all subtypes based on the half-maximal inhibitory concentration (IC50) available in the Genomics of Drug Sensitivity in Cancer database for each sample [25].

### Analysis of genomic mutations, CNAs, and microsatellite instability in CRC subtypes

Gene somatic mutation data (MAF files) containing the mutation profile of the CRC cohort were obtained from the TCGA database. The “maftools” package in R was used to analyze gene mutations among CRC subtypes [26]. Additionally, the copy number data of GISTIC_2.0 for the CRC cohort from the Genomic Data Commons (https://gdc.cancer.gov/) and chosen “*SNP6 GRCh38 Remapped Probeset File for Copy Number Variation Analysis*” as the reference file. Then, gistic scores for each sample were calculated by the freely available GenePattern software (http://cloud.genepattern.org/). Tumor mutation burden (TMB) is the total number of somatic cell-encoded mutations associated with the emergence of neoantigens that trigger antitumor immunity [27]. The TMB was measured according to tumor-specific mutated genes [28].

Microsatellite instability (MSI) is a genetic phenomenon of somatic polymorphisms in microsatellite length [29]. There is growing evidence that MSI is a recurrent somatic abnormality in several human cancers and was found in 13% of colorectal adenocarcinomas [30]. We obtained the mutation information of CRC cohort from the TCGA database, calculated the microsatellite instability scores of the samples based on the MANTIS (Microsatellite Analysis for Normal-Tumor InStability) method, as previously described [31]. Subsequently, we compared the differences in the subtypes based on the scores of the samples.

### Correlation between ferroptosis-associated CRC subtypes and clinical parameters

To evaluate whether the subtypes classified according to FRG expression profiles could serve as independent prognostic compared with other conventional clinical features (age, sex, TNM stage, tissue or organ of origin, and body mass index (BMI)) in patients with CRC, Pearson’s correlation analysis was performed using the “RCircos” package in R [17].

### Statistical analysis

The Kaplan–Meier survival analysis method with log-rank test was used to assess the differences in OS among subtypes. All statistical analyses were conducted using R software. Unless otherwise stated, values with *P* < 0.05 were considered statistically significant.

## Results

### Identification of ferroptosis-associated molecular subtypes of CRC

**Fig. 1:**
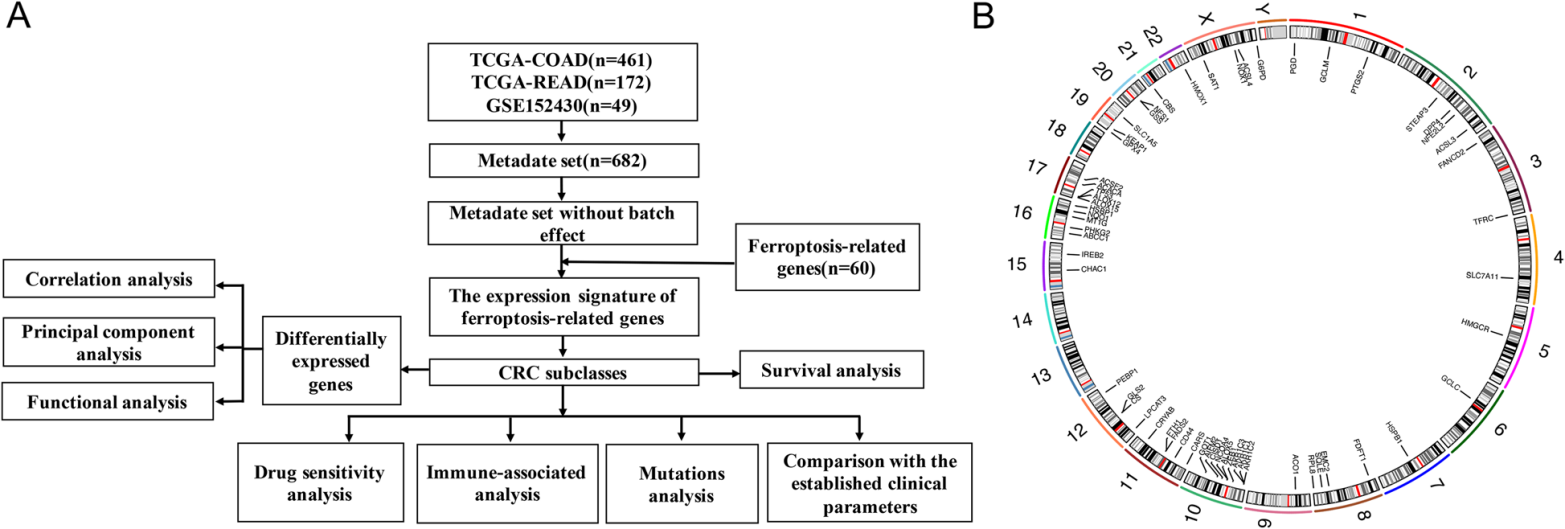
**A** Flow chart depicting the study design. **B** A Circos plot showing the distribution of ferroptosis-related genes in human chromosomes

The flow chart of this study design is shown in Fig. 1A. A total of 461 patients with colon adenocarcinoma (COAD) and 172 patients with rectum adenocarcinomas (READ) from the TCGA database and 49 patients with COAD from the GEO database were finally included in the study. The TCGA and GEO datasets were merged, and the ComBat method was applied to remove the batch effect. Next, we extracted the expression profiles of FRGs and found 59 FRGs in CRC tissues. The distribution of FRGs is depicted in a Circos plot, as shown in Fig. 1B.

**Fig. 2:**
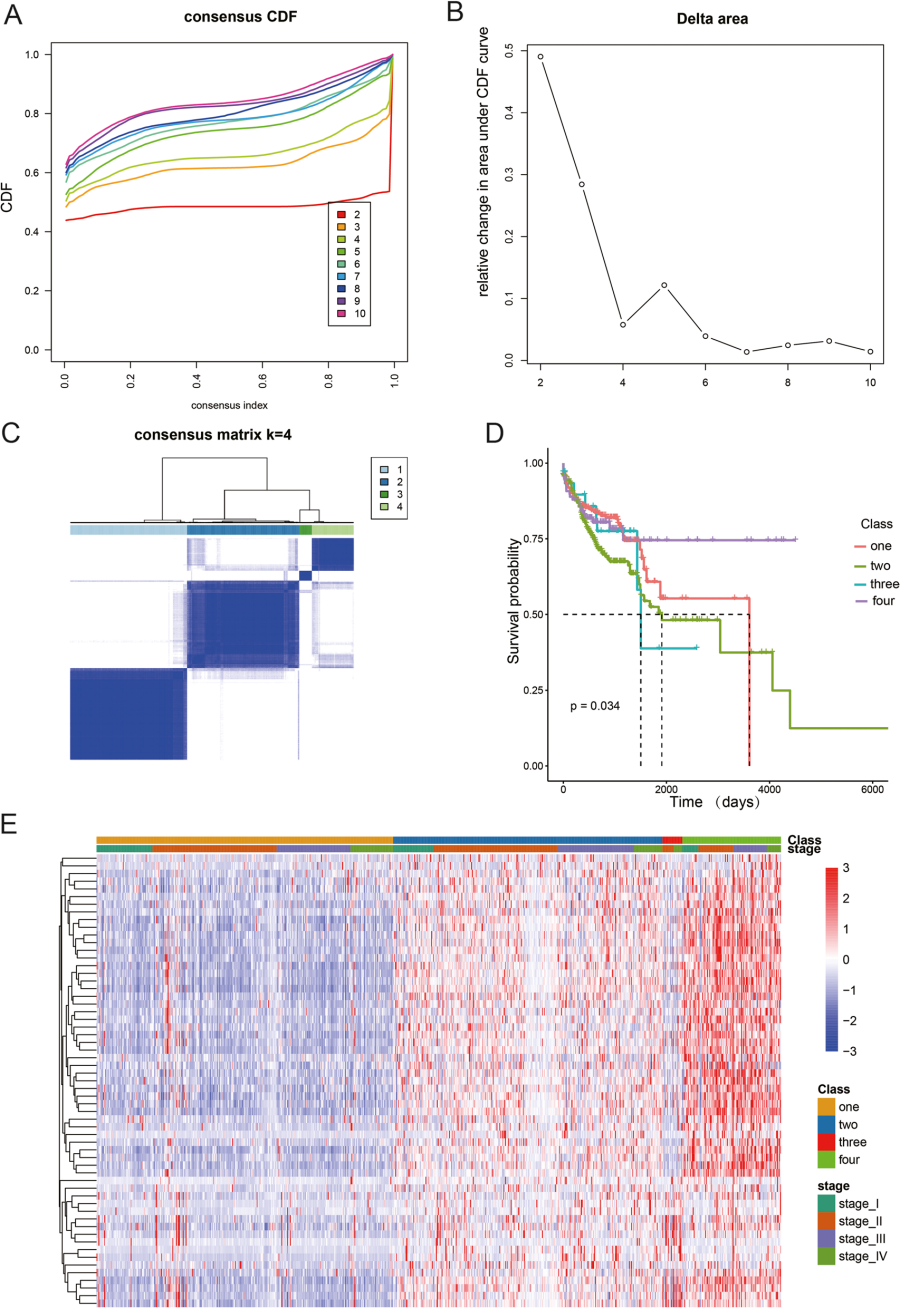
**A** The consensus among clusters for each category number k. **B** Delta area curve of consensus clustering. **C** Consensus clustering of colorectal carcinoma (CRC) samples with *k* = 4. **D** Overall survival curves of patients with CRC in each ferroptosis-associated subtype. Statistically significant differences were determined by the log-rank test. **E** A heatmap showing the correlation between the expression of ferroptosis-related genes (FRGs) and the tumor stage of the patients in each ferroptosis-associated CRC subtype. Different colors indicate different stages of CRC. The expression of FRGs is also indicated by different colors; red denotes high expression or upregulation, and green denotes low expression or downregulation

**Fig. 3:**
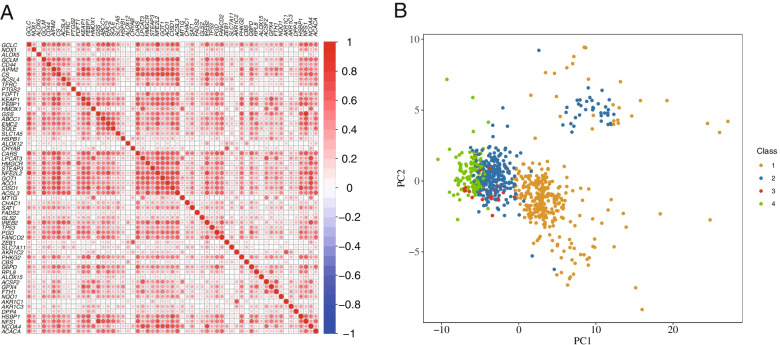
**A** A heatmap describing the co-expression of ferroptosis-related genes (FRGs). **B** Principal component analysis suggested the stratification of CRC samples into four subtypes based on the expression profile of FRGs.

By applying consensus clustering on the gene expression profile of the abovementioned 59 FRGs on the metadata set 682 CRC samples, four resulting clusters were defined: C1, C2, C3, and C4 (Fig. 2A, B). Additionally, the high similarity was observed in the gene expression pattern within each subtype based on the consensus matrix at *k* = 4 (Fig. 2C). Cox regression analysis indicated significant differences between the OS of patients with CRC with the four subtypes in the metadata set (Fig. 2D, *P* = 0.034). Next, we generated a heatmap demonstrating the relationship between the tumor stage and 59 DEGs (Fig. 2E). To validate the assignment of the ferroptosis-associated subtypes, we performed Pearson’s correlation analysis of 59 DE-FRGs, and the results showed that FRGs were co-expressed in CRC (Fig. 3A). Then, PCA was performed to compress the metadata set into a lower-dimensional dataset, and we found that the subtype designations were largely concordant with the two-dimensional PCA distribution patterns (Fig. 3B). Hence, it was reasonable to classify CRC into four subtypes based on the expression profiles of FRGs.

### Transcriptome characteristics in ferroptosis-associated CRC subtypes

**Fig. 4:**
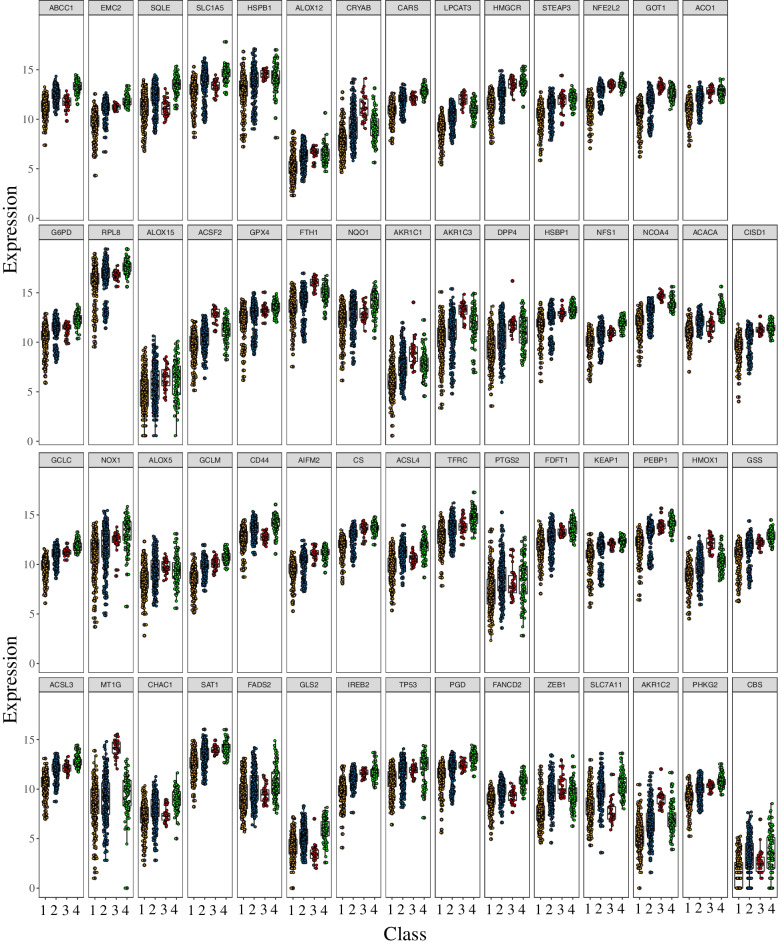
The differential expression of 59 ferroptosis-related genes in different ferroptosis-associated colorectal carcinoma (CRC) subtypes

To further characterize the DE-FRGs in the four subtypes, we performed ANOVA for differential gene expression analysis. Differences in gene expression were considered statistically significant if the BH-adjusted *P*-value was < 0.05. The results indicated that all 59 FRGs were differentially expressed in all four subtypes (Fig. 4).

**Fig. 5:**
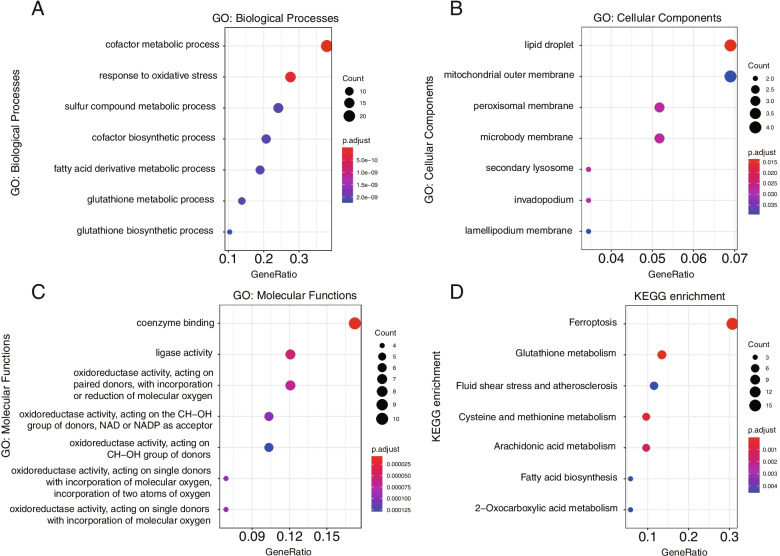
Functional enrichment analysis of differentially expressed ferroptosis-related genes in different subtypes

To elucidate the biological functions and pathways of the 59 DEGs in CRC, GO enrichment analysis was conducted using the “clusterProfiler” package. Significantly enriched biological process GO terms are shown in Fig. 5. GO enrichment analysis showed that these FRGs were significantly associated with biological processes, including metabolic processes and oxidative stress, and KEGG pathway analysis revealed that they were associated with metabolic pathways such as iron-dependent cell death and glutathione metabolism.

### Characteristics of the tumor-immune microenvironment among ferroptosis-associated CRC subtypes

**Fig. 6:**
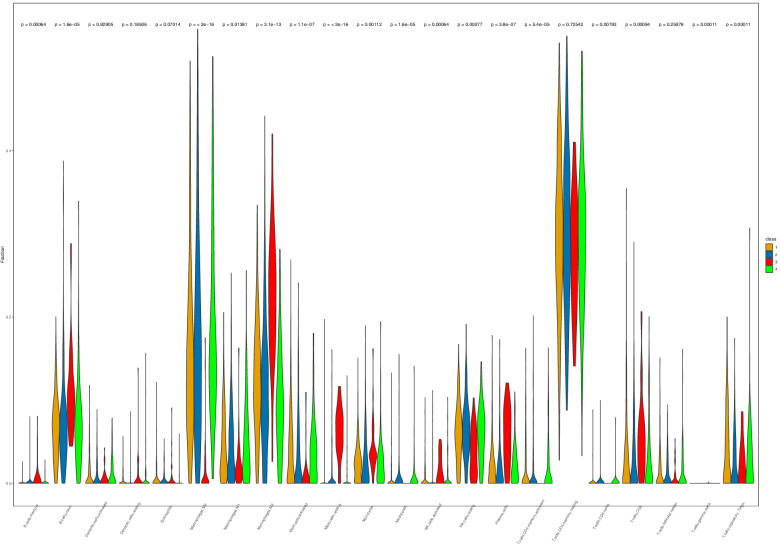
Differences in immune cell infiltration in ferroptosis-associated colorectal carcinoma (CRC) subtypes

**Fig. 7:**
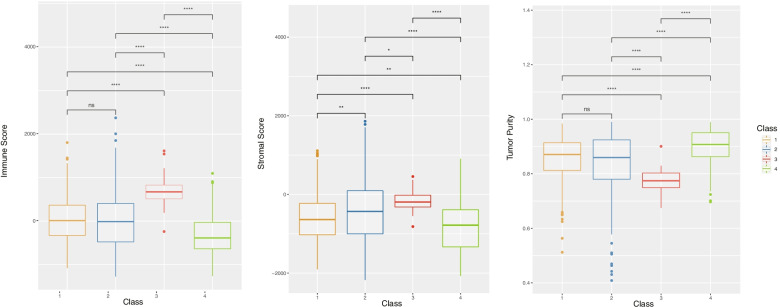
Immune scores, stromal scores, and tumor purity in ferroptosis-associated colorectal carcinoma (CRC) subtypes. *ns*, no significance; *P < 0.05, **P < 0.01, and ****P < 0.0001

To uncover the immune heterogeneity among the four subtypes, we used immune-related tools. Using the CIBERSORT method, we evaluated the differences in the immune infiltration of 22 immune cells among the four subtypes in patients with CRC. As illustrated in Fig. 6, the infiltration pattern of 18 immune cells was different among the four subtypes. Compared with the other three subtypes, the C3 subtype had a higher abundance of seven immune cell populations (B cells, M2 macrophages, resting mast cells, monocytes, natural killer cells, plasma cells, and CD8^+^ T cells). In addition, the C3 subtype also exhibited low infiltration of M1 macrophages, activated mast cells, neutrophils, T follicular helper cells, and T regulatory cells (Fig. 6). We further investigated stromal and immune scores based on the ESTIMATE method. The results indicated that the C3 subtype had the highest immune scores, and the C4 subtype had the lowest immune scores (C3 > C1/C2 > C4). Similarly, the C3 subtype had the highest stromal scores, and the C4 subtype had the lowest stromal scores (C3 > C2 > C1 > C4). In contrast, the C2 subtype showed the lowest tumor purity, while the C4 subtype showed the highest tumor purity (C4 > C1/C2 > C3) (Fig. 7).

### Assessment of immunotherapeutic response in ferroptosis-associated CRC subtypes

**Fig. 8:**
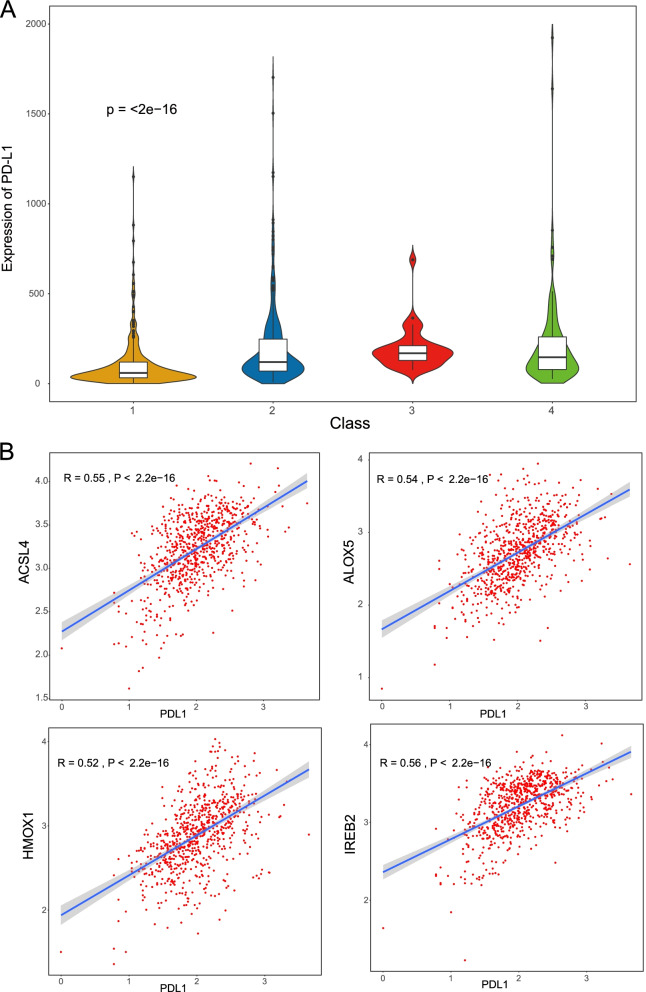
**A** The mRNA expression of programmed cell death ligand 1 (*PD-L1*) in ferroptosis-associated subtypes of patients with colorectal carcinoma (CRC). **B** The correlation between *PD-L1* expression and the expression of *ACSL4*, *ALOX5*, *HMOX1*, and *IREB2* genes

**Fig. 9:**
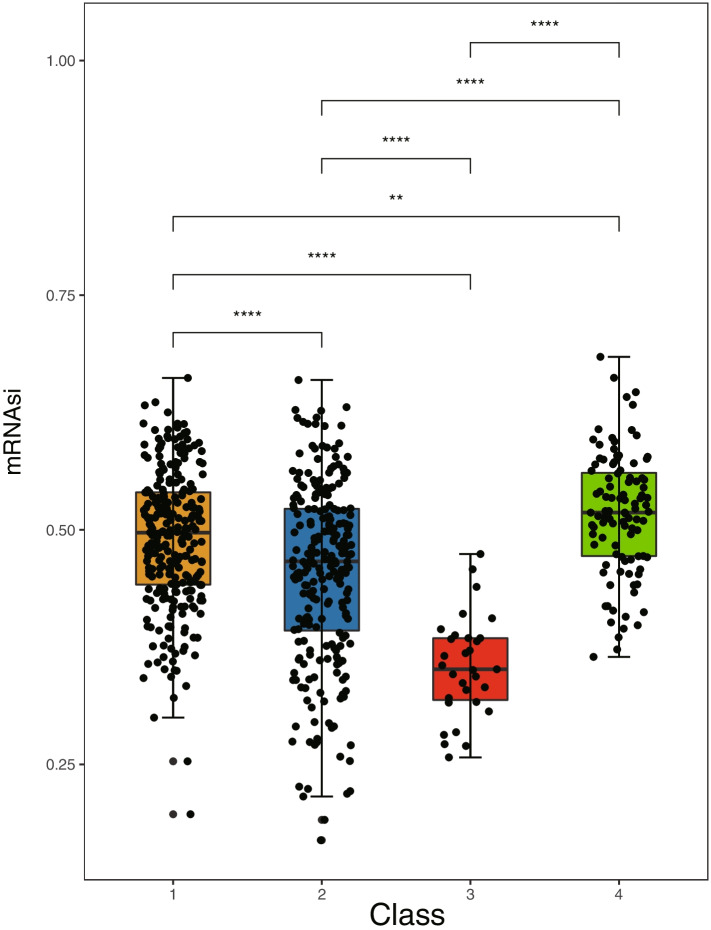
Differences in the stemness index among four ferroptosis-associated colorectal carcinoma (CRC) subtypes. **P < 0.01 and ****P < 0.0001

Immunotherapy has emerged as a promising strategy for the treatment of many cancers. In this study, we investigated the expression of PD-L1, which is a pivotal immunomodulator. As shown in Fig. 8A, the expression of PD-L1 was different in each subtype, and patients with the C3 subtype had a higher expression of PD-L1 than those with other subtypes. In addition, we investigated the correlation between PD-L1 expression and FRGs in patients with CRC. The results revealed that 49 FRGs were significantly correlated with PD-L1 expression. We only displayed the associated genes (*ACSL4*, *ALOX5*, *HOMX1*, and *IREB2*) with absolute values of the Pearson correlation coefficient > 0.5 and *P* values < 0.01 (Fig. 8B), and the correlation results for the remaining genes are shown in Table S3. Cancer stem cells are known to play a critical role in the growth, metastasis, and recurrence of tumors. Recent studies have reported that higher stemness indices are associated with limited antitumor immune responses and decreased PD-L1 expression [32]. In this study, we applied the OCLR algorithm to calculate stemness indices of the four CRC subtypes. As shown in Fig. 9, there was a significant difference in the stemness index among the subtypes, and the C3 subtype demonstrated the lowest values for stemness indices (C4 > C1 > C2 > C3). Thus, these data support our analysis that patients with the C3 subtype may have a better prognosis and may respond positively to immunotherapy.

### Different susceptibility to antitumor drugs among ferroptosis-associated CRC subtypes

**Fig. 10:**
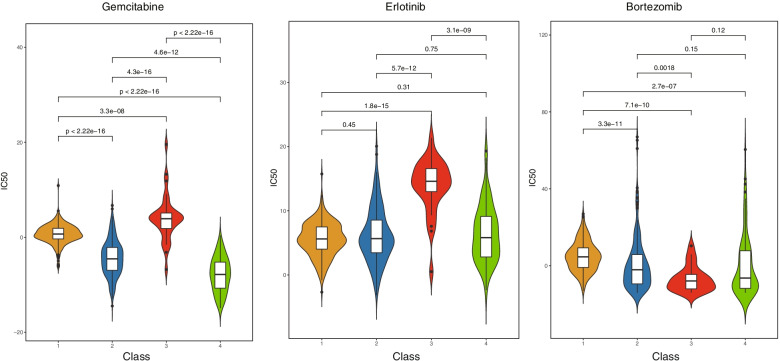
Differences in antitumor drug sensitivity among ferroptosis-associated subtypes in patients with colorectal carcinoma (CRC)

By using the “pRRophetic” package in R, we further explored the association between the CRC subtypes and sensitivity toward antitumor drugs. We found that six compounds were screened out for significant differences in the estimated IC50 among the four subtypes. Among them, the sensitivity to gemcitabine was significantly different among the four subtypes (*P* < 0.0001). The C3 subtype was significantly associated with erlotinib resistance. The sensitivity to bortezomib was also significantly different among the four subtypes (Fig. 10). Furthermore, the sensitivity of the CRC subtypes to other drugs such as cytarabine, dasatinib, and sorafenib was explored, and significant differences were observed among the subtypes (Supplementary Fig. S2). These data demonstrated that the ferroptosis-associated CRC subtypes might be used for the implementation of precision therapeutic approaches in the future.

### Differences in mutations, CNAs, and MSI in ferroptosis-associated CRC subtypes

**Fig. 11:**
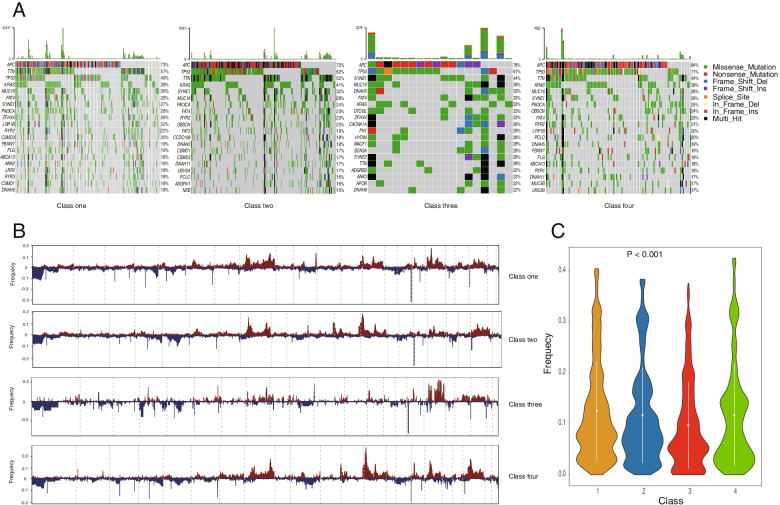
**A** A comprehensive oncoprint depicting the mutation status of genes among ferroptosis-associated colorectal carcinoma (CRC) subtypes. **B** Gene mutation maps among ferroptosis-associated colorectal carcinoma (CRC) subtypes. **C** A violin plot of the frequency of copy number alterations (CNAs) in ferroptosis-associated colorectal carcinoma (CRC) subtypes

The success of targeted therapies and immunotherapy is largely dependent on the cancer genomic landscape. We further evaluated the differences in gene mutations and CNAs among the four subtypes. The results revealed that distinct subtypes had different mutational characteristics. For example, 49% of C1 samples, 62% of C2 samples, and 71% of C4 samples had *TP53* mutation (Fig. 11A). Next, we obtained the CNA frequencies of different subtypes according to the GenePattern software package, and the results indicated a significant difference in the frequency of CNAs among the four subtypes (Fig. 11B, C). We then calculated TMB scores based on TCGA somatic mutation data. The TMB in C4 is higher than that in C2 subtypes (Supplementary Fig. S3A, *P* = 0.018). Furthermore, we employed the tool, MANTIS (Microsatellite Analysis for Normal Tumor InStability), for detecting the MSI status from the TCGA dataset. We found that MSI in C1 was all significantly different compared with that in C2, C3, as well as C4 (*P* < 0.05) (Supplementary Fig. S3B). Taken together, these data imply that unique genomic features and biological heterogeneity exist among the four subtypes and that the classification of CRC subtypes based on ferroptosis-related genes is robust.

### Ferroptosis-associated subtypes can predict CRC prognosis independently of patients’ clinical characteristics

**Fig. 12:**
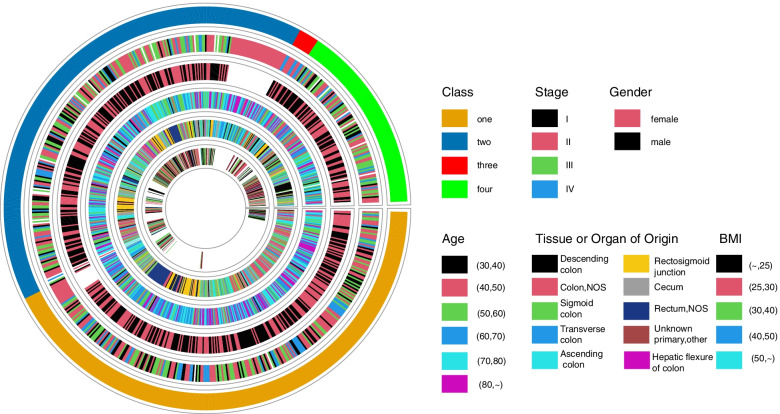
A Circos plot describing the comparison of ferroptosis-associated colorectal carcinoma (CRC) subtypes with the distribution of conventional clinical parameters (from outside to inside: the first circle indicates the ferroptosis-associated molecular subtypes (Class), the second circle indicates the stage of tumor (Stage), the third circle indicates gender (Gender), the fourth circle indicates age (Age), the fifth circle indicates the site of tumor occurrence (Tissue or Organ of Origin), and the sixth circle indicates the body mass index (BMI))

Next, we aimed to elucidate whether the CRC subtypes based on FRGs could be used as independent predictors for the prognosis of patients with CRC compared with other conventional clinical parameters. By integrating clinical data downloaded from patients with COAD and READ in TCGA with the clinical information obtained from GEO, we obtained the clinical information of the metadata set. By plotting a Circos plot, we compared the association of ferroptosis-associated CRC subtypes with the tumor stage, age, sex, tissue or organ of origin, and BMI of patients. As shown in Fig. 12, ferroptosis-associated CRC subtypes did not overlap with other clinical parameters in the prognosis of CRC. Hence, the ferroptosis classification may serve as a potential molecular subtype that can provide an important prognostic and therapeutic value in CRC.

## Discussion

Ferroptosis is a new type of iron-dependent programmed cell death, which is different from other types of cell death. Recent studies have indicated that the correlation between ferroptosis and cancer is complex and that ferroptosis induction is considered a novel approach for cancer therapy [33–35]. However, the systematic analysis of CRC is yet to be elucidated. In this study, to classify CRC samples based on the expression profiles of FRGs, we analyzed the gene expression profile of CRC samples in TCGA and GEO datasets after removing the batch effect from the data. The consensus clustering based on our large sample size indicated that our transcriptome classification of CRC was robust. Then, we screened 59 FRGs that could be used to cluster patients with CRC into four groups, with significant differences in clinicopathologic parameters and prognosis. Next, we explored the transcriptome features, immune cell characteristics, and drug sensitivity of the subtypes.

Recently, a few studies have indicated that several ferroptosis-related genes such as TP53, GPX4, SLC7A11, and ACSL4 could regulate drug-induced ferroptosis in CRC; however, the relationship between their expressions with the clinical outcome of patients with CRC remains unknown. In this study, we found that most FRGs (59/60) were differentially expressed in CRC tissues, and each subclass presented different prognoses. These results significantly demonstrated the potential role of ferroptosis in CRC.

The tumor microenvironment and immune cell infiltration are reported to be correlated with cancer prognosis [36]. Moreover, some studies conducted over the past 5 years have revealed that ferroptosis is closely associated with antitumor immunity [37]. In this study, we investigated the relationship between ferroptosis-associated molecular subtypes of CRC and immune markers, including immune score, tumor purity, and immune cell infiltration. As expected, the ferroptosis-associated subtypes could successfully distinguish patients with CRC based on the above makers, thus implying the credibility of this molecular classification in evaluating the immune response. Moreover, the C3 subtype had the higher immune and stromal scores and the lowest tumor purity. These data suggest that the C3 subtype has a high heterogeneity and that patients with this subtype may have refractory cancer. In recent years, immune checkpoint inhibitors have been successfully applied to the treatment of various cancers. The expression of PD-L1 is a commonly used marker for predicting the efficacy of immunotherapy. Patients with high levels of PD-L1 in tumor tissues who received PD-1/PD-L1 inhibitors have better survival outcomes compared with those who did not receive this treatment [38, 39]. Thus, we investigated the expression of the immune checkpoint molecule PD-L1 and analyzed the correlation between the expression of PD-L1 and FRGs. The results showed that the expression of PD-L1 was significantly different among the four subgroups, and the expression of PD-L1 was significantly correlated with that of several FRGs. These results further indicated that ferroptosis was significantly correlated with the efficacy of immunotherapy. Cancer stemness has been reported to be associated with suppressed immune responses across several cancer types. As expected, we found significant differences in the stemness index among subtypes in our study. Based on the above evidence, we hypothesize that the molecular subtypes based on FRGs may serve as reliable immune-related biomarkers for the prognosis and management of cancer.

Gene mutations are known to be associated with treatment resistance. In our study, the genomic analysis revealed a distinct mutation and copy number variation landscape among subtypes. The results indicated that distinct subtypes tended to have different mutation frequencies in each gene. TMB indicates the total number of somatic mutations. Several studies have suggested that TMB might be a useful biomarker to estimate the new antigen load of a tumor [40]. We found that TMB was significantly different only in C2 and C4. Additionally, CRCs display a heterogeneous immune landscape based on their microsatellite status, and recent studies revealed that MSI was an independent biomarker to predict the efficacy of immune checkpoint inhibitors in CRC [41, 42].

We found there were significant differences in MSI between C1 and other subtypes, and C3 had a higher MSI score. Collectively, these data demonstrated that the identified ferroptosis-associated subtypes were closely associated with gene alterations; however, the correlations between ferroptosis and somatic mutations need to be further studied.

Next, we screened the potential compounds and tested the sensitivity of antitumor drugs among subtypes. Interestingly, the classical antitumor agents currently administered for clinical treatment in CRC such as fluorouracil, irinotecan, oxaliplatin, and cetuximab did not display differences in the four subtypes, and these results may imply that whether these agents are linked to ferroptosis in CRC remains to be explored in the future. The results showed significant differences in drug sensitivity among the four subtypes, suggesting the involvement of different mechanisms in CRC among the four subtypes.

Then, we compared the classification with the distribution of conventional clinical parameters. The results revealed that the molecular classification of ferroptosis based on FRGs did not significantly overlap with the classification of other clinical parameters in CRC.

In the present study, we classified CRC based on the molecular signature of FRGs for the first time. However, we must acknowledge that our present work presents some limitations. First, our classification needs to be validated on datasets with larger sample size. Second, our ferroptosis-associated classification needs to be validated in a multicenter clinical sample before future application. Additionally, further experiments are needed to understand the difference in the mechanisms associated with cancer development among the four subtypes of CRC.

## Conclusions

In this study, we established classified CRC into four subtypes based on FRG expression profiles. These subtypes may have their unique gene expression patterns. Our study may provide valuable insights into the study of ferroptosis signatures in CRC and the development of strategies for personalized treatment and prognosis prediction.

## Supplementary Information

**Additional file 1: Table S1.** Clinical characteristics of TCGA and GEO sets. N/A, not available; BMI, body mass index. **Table S2.** The 60 ferroptosis-related genes. **Table S3.** Correlations between PD-L1 and 49 ferroptosis-associated genes. **Supplementary Fig. S1.** Principal component analysis showed the distribution of samples in GEO and TCGA datasets. **Supplementary Fig. S2.** Differences in antitumor drug (sorafenib(a), dasatinib(b) and cytarabine(c)) sensitivity in ferroptosis subclasses of CRC patients. **Supplementary Fig. S3.** (A) Differences in the tumor mutation burden (TMB) in ferroptosis-associated subtypes in patients with colorectal carcinoma (CRC). (B) Differences in microsatellite instability (MSI) scores in ferroptosis-associated subtypes in patients with colorectal carcinoma (CRC).

## Data Availability

The data that support the findings of this study are available from the corresponding author upon reasonable request.
