# Supplementary material for: Classification of colorectal carcinoma subtypes based on ferroptosis-associated molecular markers
Source: World J Surg Oncol. 2022 Apr 12;20:117. doi: 10.1186/s12957-022-02575-5 (PMC9004151; doi:10.1186/s12957-022-02575-5)
Supplement: Supplementary file 1 — Additional file 1: Table S1. Clinical characteristics of TCGA and GEO sets. N/A, not available; BMI, body mass index. Table S2. The 60 ferroptosis-related genes. Table S3. Correlations between PD-L1 and 49 ferroptosis-associated genes. Supplementary Fig. S1. Principal component analysis showed the distribution of samples in GEO and TCGA datasets. Supplementary Fig. S2. Differences in antitumor drug (sorafenib(a), dasatinib(b) and cytarabine(c)) sensitivity in ferroptosis subclasses of CRC patients. Supplementary Fig. S3. (A) Differences in the tumor mutation burden (TMB) in ferroptosis-associated subtypes in patients with colorectal carcinoma (CRC). (B) Differences in microsatellite instability (MSI) scores in ferroptosis-associated subtypes in patients with colorectal carcinoma (CRC). [file 12957_2022_2575_MOESM1_ESM.docx]

**Supplementary Materials**

| Table S1 clinical characteristics of TCGA and GEO sets. N/A, not available; BMI, body mass index. | | |
| --- | --- | --- |
| Variable | GSE152430(n) | TCGA(n) |
| Gender |  |  |
| male | N/A | 335 |
| female | N/A | 294 |
| Age(years) |  |  |
| >80 | 17 | 101 |
| 70-80 | 19 | 173 |
| 60-70 | 9 | 177 |
| 50-60 | 3 | 101 |
| 40-50 | 1 | 60 |
| <40 | 0 | 17 |
| Stage |  | 109 |
| I | N/A |  |
| II | 49 | 229 |
| III | N/A | 181 |
| IV | N/A | 90 |
| NA |  | 24 |
| Tissue or organ of origin |  |  |
| Descending colon | 2 | 17 |
| Cecum | N/A | 90 |
| Colon, NOS | N/A | 110 |
| Ascending colon | 17 | 92 |
| Hepatic flexure of colon | N/A | 12 |
| Rectosigmoid junction | N/A | 79 |
| Rectum, NOS | N/A | 109 |
| Sigmoid colon | 9 | 131 |
| Splenic flexure of colon | N/A | 5 |
| Transverse colon | 21 | 21 |
| Unknown primary site, other | N/A | 3 |
| BMI |  |  |
| <25 | N/A | 99 |
| 25-30 | N/A | 117 |
| 30-40 | N/A | 74 |
| 40-50 | N/A | 12 |
| >50 | N/A | 3 |

| Table S2. The 60 ferroptosis-related genes. | | | | |
| --- | --- | --- | --- | --- |
| Gene symbol | Name | | | |
| ACSL4 | acyl-CoA synthetase long-chain family member 4 | | | |
| AKR1C1 | aldo-keto reductase family 1 member C1 | | | |
| AKR1C2 | aldo-keto reductase family 1 member C2 | | | |
| AKR1C3 | aldo-keto reductase family 1 member C3 | | | |
| ALOX15 | [arachidonate 15-lipoxygenase](https://www.ncbi.nlm.nih.gov/gene/246) | | | |
| ALOX5 | [arachidonate 5-lipoxygenase](https://www.ncbi.nlm.nih.gov/gene/240) | | | |
| ALOX12 | [arachidonate 12-lipoxygenase](https://www.ncbi.nlm.nih.gov/gene/240) | | | |
| ATP5MC3 | ATP synthase membrane subunit c locus 3 | | | |
| CARS | cysteinyl tRNA synthetase | | | |
| CBS | cystathion ine beta synthase | | | |
| CD44 | CD44 molecule | | | |
| CHAC1 | ChaC glutathione- specific gamma-glutamyl cyclotransferase 1 | | | |
| CISD1 | CDGSH iron sulfur domain 1 | | | |
| CS | citrate synthase | | | |
| DPP4 | dipeptidyl-dippeptidase-4 | | | |
| FANCD2 | Fanconi anemia comple mentation group D2 | | | |
| GCLC | glutamate-cysteine ligase catalytic subunit | | | |
| GCLM | glutamate-cysteine ligase modifier subunit | | | |
| GLS2 | glutaminase 2 | | | |
| GPX4 | glutathio ne peroxidase 4 | | | |
| GSS | glutathione synthetase | | | |
| HMGCR | 3-hydroxy-3- methylglutaryl-CoA reductase | | | |
| HSPB1 | heat shock protein beta 1 | | | |
| CRYAB | heat shock protein beta 5 | | | |
| LPCAT3 | lysophosp hatidylcholine acyltransferase 3 | | | |
| MT1G | metallothionein-1G | | | |
| NCOA4 | nuclear receptor coactiva tor 4 | | | |
| PTGS2 | prostagla ndin-endoperoxide synthase 2 | | | |
| RPL8 | ribosomal protein L8 | | | |
| SAT1 | spermidine/spermine N1-acetyltra nsferase 1 | | | |
| SLC7A11 | solute carrier family 7 member 11 | | | |
| FDFT1 | farnesyl-diphosphate farnesyltransferase 1 | | | |
| TFRC | transferrin receptor | | | |
| TP53 | tumor protein 53 | | | |
| EMC2 | ER membrane protein complex subunit 2 | | | |
| AIFM2 | apoptosis inducing factor mitochondria associated 2 | | | |
| PHKG2 | phospho rylase kinase, g2 | | | |
| HSBP1 | heat-shock 27-k Da protein 1 | | | |
| ACO1 | aconitase 1 | | | |
| FTH1 | ferritin heavy chain 1 | | | |
| STEAP3 | six-transm embrane epithelial antigen of prostate 3 | | | |
| NFS1 | cysteine desulfurase | | | |
| ACSL3 | acyl-CoA synthetase long-chain family member 3 | | | |
| ACACA | Acetyl-CoA carboxylase alpha | | | |
| PEBP1 | phosphatidy lethanolamine-binding protein 1 | | | |
| ZEB1 | zinc finger E-box-binding homeobox 1 | | | |
| SQLE | squalene monooxygenase | | | |
| FADS2 | fatty acid desaturase 2/acyl-CoA 6-desaturase | | | |
| NFE2L2 | nuclear factor, erythroid 2 like 2 | | | |
| KEAP1 | kelch-like ECH- associated protein 1 | | | |
| NQO1 | quinone oxidoreductas e-1 | | | |
| NOX1 | NADPH oxidase 1 | | | |
| ABCC1 | ATP binding cassette subfamily C member 1 | | | |
| SLC1A5 | solute carrier family 1 member 5 | | | |
| GOT1 | glutamic-oxa loacetic transaminase 1 | | | |
| G6PD | glucose-6-phosphate dehydrogenas e | | | |
| PGD | phosphoglycerate dehydrogenas e | | | |
| IREB2 | iron response element-binding protein 2 | | | |
| HMOX1 | heme oxygenase 1 | | | |
| ACSF2 | acyl-CoA synthetase family member 2 | | | |
| Table S3 Correlations between PD-L1 and 49 ferroptosis-associated genes. | | | |  |
| Ferroptosis- associated genes | | R (Pearson’s correlation) | P-value |  |
| ABCC1 | | 0.32 | <2.2e-16 |  |
| ACACA | | 0.35 | <2.2e-16 |  |
| ACO1 | | 0.45 | <2.2e-16 |  |
| ACSL3 | | 0.45 | <2.2e-16 |  |
| ACSL4 | | 0.55 | <2.2e-16 |  |
| AIFM2 | | 0.21 | 9.9e−09 |  |
| AKR1C1 | | 0.22 | 1.4e-09 |  |
| AKR1C2 | | 0.17 | 3.4e-06 |  |
| ALOX12 | | 0.39 | <2.2e-16 |  |
| ALOX5 | | 0.54 | <2.2e-16 |  |
| CBS | | 0.15 | 2.2e-05 |  |
| CD44 | | 0.47 | <2.2e-16 |  |
| CHAC1 | | 0.17 | 2.6e-06 |  |
| CISD1 | | 0.3 | <2.2e-16 |  |
| CRYAB | | 0.3 | <2.2e-16 |  |
| CS | | 0.37 | <2.2e-16 |  |
| DPP4 | | 0.36 | <2.2e-16 |  |
| EMC2 | | 0.4 | <2.2e-16 |  |
| FADS2 | | 0.24 | 2.3e-11 |  |
| FANCD2 | | 0.42 | <2.2e-16 |  |
| FDFT1 | | 0.24 | 1.8e-11 |  |
| FTH1 | | 0.21 | 5.5e-09 |  |
| G6PD | | 0.16 | 9.4e-06 |  |
| GCLC | | 0.35 | <2.2e-16 |  |
| GCLM | | 0.44 | <2.2e-16 |  |
| GLS2 | | 0.2 | 4.6e-08 |  |
| GOT1 | | 0.31 | <2.2e-16 |  |
| HMGCR | | 0.36 | <2.2e-16 |  |
| HMOX1 | | 0.52 | <2.2e-16 |  |
| HSBP1 | | 0.23 | 2.3e-10 |  |
| IREB2 | | 0.56 | <2.2e-16 |  |
| KEAP1 | | 0.15 | 6.2e-05 |  |
| LPCAT3 | | 0.38 | <2.2e-16 |  |
| MT1G | | 0.14 | 0.00019 |  |
| NCOA4 | | 0.47 | <2.2e-16 |  |
| NFE2L2 | | 0.43 | <2.2e-16 |  |
| NFS1 | | 0.13 | 0.00033 |  |
| NQO1 | | 0.14 | 0.00018 |  |
| PEBP1 | | 0.19 | 3e-07 |  |
| PGD | | 0.24 | 2.2e-11 |  |
| PHKG2 | | 0.21 | 7.8e-09 |  |
| PTGS2 | | 0.56 | <2.2e-16 |  |
| SAT1 | | 0.46 | <2.2e-16 |  |
| SLC7A11 | | 0.45 | <2.2e-16 |  |
| SQLE | | 0.18 | 6.3e-07 |  |
| STEAP3 | | 0.2 | 2e-08 |  |
| TFRC | | 0.36 | <2.2e-16 |  |
| TP53 | | 0.19 | 1.3e-07 |  |
| ZEB1 | | 0.54 | <2.2e-16 |  |

Supplementary Fig.S1 Principal component analysis showed the distribution of samples in GEO and TCGA datasets.

Supplementary Fig.S2 Differences in antitumor drug (sorafenib(a), dasatinib(b) and cytarabine(c)) sensitivity in ferroptosis subclasses of CRC patients.

Supplementary Fig.S3 (A)Differences in the tumor mutation burden (TMB) in ferroptosis-associated subtypes in patients with colorectal carcinoma (CRC). (B) Differences in microsatellite instability (MSI) scores in ferroptosis-associated subtypes in patients with colorectal carcinoma (CRC).
